# Intra-coronary physiology in contemporary percutaneous coronary intervention and anginal therapy with a focus on microvascular disease

**DOI:** 10.3389/fcvm.2023.1255643

**Published:** 2023-11-29

**Authors:** Zaheer Alisiddiq, Harish Sharma, James Cotton, Lampson Fan

**Affiliations:** ^1^ Department of Cardiology, New Cross Hospital, The Royal Wolverhampton NHS Trust, Wolverhampton, United Kingdom; ^2^ The Institute of Cardiovascular Sciences, College of Medical and Dental Sciences, University of Birmingham, Edgbaston, Birmingham, United Kingdom; ^3^ Department of Cardiology, New Cross Hospital, Wolverhampton, United Kingdom; ^4^ Department of Molecular and Clinical Medicine, Faculty of Science and Engineering, Research Institute of Healthcare Science, University of Wolverhampton, Wolverhampton, United Kingdom

**Keywords:** coronary physiology, microvascular disease, angina, coronary angiography, coronary artery disease

## Abstract

Coronary physiological measurements have transformed the treatment of coronary artery disease (CAD), with increasing evidence supporting the use of pressure wire guided revascularisation. Advances in microvascular assessment have enabled clinicians to discern angina aetiology even in patients without obstructive epicardial coronary artery disease, paving the way for more effective tailored therapy. In this article, the authors will examine pressure wire indices, their role in influencing clinical outcomes and future directions.

## Introduction

Currently more than ten different modalities exist for the assessment of coronary physiology. Despite the wealth of evidence and technology available, however, most lesions are still treated based on visual assessment. In a survey from 2014, 71% of cases were managed following angiography alone whereas fractional flow reserve (FFR) was utilised in 21% (1). Despite changes in guidelines, seven years from the 2014 survey, functional assessment increased to merely 31% (2). In this review we will examine the different physiological assessment modalities, their impact on planning and decision making in contemporary percutaneous coronary intervention (PCI and their impact on clinical outcomes. In addition we discuss new developments and future directions in this important area.

## Modalities of coronary physiology

### Pressure wire coronary physiology

#### Hyperaemic pressure ratio

**Figure 1:**
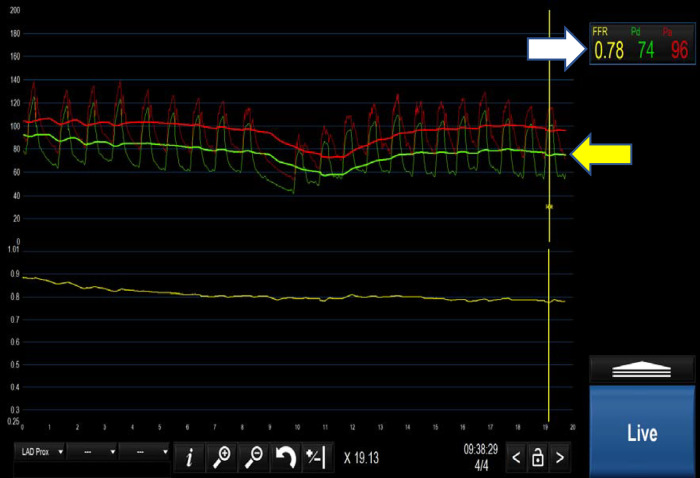
Positive FFR <0.80 (indicated by white arrow). Yellow arrow demonstrates the separation of the distal coronary pressure (green waveform) from the aortic pressure (red waveform) during hyperaemia induced by intravenous adenosine.

Fractional flow reserve (FFR) is the most well-known and established method for the assessment of coronary physiology; it is considered widely to be the gold standard. FFR is defined as the mean ratio of distal coronary pressure to aortic pressure during maximal hyperaemia (typically induced by adenosine see Figure 1). In essence, FFR represents the percentage by which an epicardial coronary stenosis impairs myocardial flow.

The assessment of multiple lesions in one artery, or simply tandem lesions is complex, and many pressure wire systems try to address this difficult area by allowing for a “pull back” recording, either during maximal hyperaemia or during measurement of the instantaneous free wave ratio (IFR). The interaction of the impact of tandem lesions of the fluid dynamics within the artery do make these assessments somewhat difficult to interpret and caution is usually applied to the results in clinical practice.

The clinical significance of FFR was initially validated against non-invasive tests in 1996 and a binary “cut-off” value of 0.75 (17) was postulated. Later, the landmark FAME I trial demonstrated clear superiority of FFR-guided PCI (using an FFR threshold of <0.80, to include a 0.05 “grey area”) compared to angiography-guided PCI in the composite end-points of death, myocardial infarction (MI) and repeat revascularisation at 1-year for patients with stable angina (18). It was also more cost effective without prolonging the procedure time (19). At 5-year follow up, this difference persisted, although it was no longer statically significant (20). Subsequently, the FAME II trial demonstrated FFR-guided PCI was superior to medical therapy, although outcomes were primarily driven by urgent revascularisation rather than death or MI (21, 22). This was further confirmed after 5 years of follow-up (23).

There is growing evidence for utilising FFR in patients with acute coronary syndrome (ACS). Several studies have demonstrated FFR can accurately determine the functional significance of non-culprit coronary lesions in ACS patients with improved clinical outcomes (24–27) In the COMPARE-Acute and DANAMI-3-PRIMULTI trials, patients with FFR-guided complete revascularisation had significantly reduced composite end-outcomes, primarily driven by reduced repeat revascularisation (26, 27). In patients with STEMI and multi vessel disease, the functional assessment of non cultprit lesions in the context of a STEMI has been questioned due to the microvasculature status in remote myocardial territories, which could effect the reliability of iFR and FFR (28). As mentioned above, numerous trials like the DANAMI-3-PRIMULTI anCOMPARE-Acute trials addressed this question and generally favored the use of coronary physiology in revascularization of the non culprit vessel (26, 27). Furthermore, in the COMPARE-Acute trial, the functional assessment was performed during the primary pci, whereas in the DINAMI-3-PRIMULTI, it was performed as a staged procedure prior to discharge. Interstingly, both demonstated the superiority of FFR-guided complete revascularization. The question then arises weather these patients should have hyperaemic or non hypeaemic assessment. The WAVE study compared iFR with FFR assessment of non culprit arteries during primary pci and demonstrated similar diagnostic yields (29).

More recently, the COMPLETE trial demonstrated significant reduction in MI and cardiovascular death with complete revascularisation in STEMI patients compared to culprit-only treatment based on angiography alone, rather than physiology (30, 31). Similarly, the FLOWER-MI trial found no difference between FFR-guided complete revascularisation compared to an angiography-guided strategy. However, this trial was under-powered due to low event rates and had wide confidence intervals for the hazard ratio of the primary endpoint (31). It is worth noting that FFR is a measurement of pressure and a surrogate for flow based on experimental conditions (30), which may contribute to suboptimal results of FFR in the post-MI setting.

A subset of patients with FFR negative lesions may be harbouring vulnerable plaques at high risk of future events [for example due to the presence of thin cap fibroatheromas (TCFA)]. This insight primarily comes from two observational studies: COMBINE OCT-FFR (32) (examining diabetic patients with predominantly stable coronary artery disease) and PECTUS-Obs (33) (examining MI patients). These studies demonstrate that up to a quarter of FFR negative lesions can be classified as having TCFA, which is associated with significantly increased major adverse clinical events. However, there is currently a lack of randomized control trials inestigating the clinical effectiveness of routine evaluation of vulnerable plaques.

#### Non-hyperaemic pressure ratios (NHPR)

The major perceived drawback of the hyperaemic pressure ratio method is the need to administer intravenous adenosine, or other hyparaemic agents. Adenosine causes microvascular hyperaemia to maintain constant microvascular resistance. In this state, the pressure across an epicardial lesion is proportional to the flow, without the influence of any microvascular dysfunction. Despite its very short half-life, adenosine is often not well tolerated. In order to avoid this problem, the concept of the measurement of non-hyperaemic pressure ratios (NHPR) was developed. These measurements derive from the ratio of distal coronary pressure (Pd) to aortic pressure (Pa) over periods in the cardiac cycle where microvascular resistance is in a steady-state or at the lowest value. A number of indices have been proposed which include measurement of mean Pd/Pa in a specific point in diastole (instantaneous wave-free period), over the entire diastolic period (diastolic hyperaemia-free ratio), or as a single lowest value over the entire cardiac cycle (resting full-cycle ratio).

**Figure 2:**
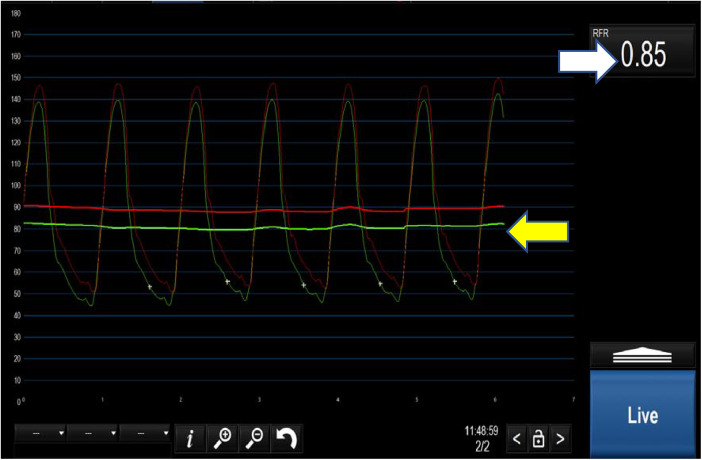
Positive iFR <0.89 (white arrow). The yellow arrow demonstrates the separation of the distal coronary pressure (green waveform) from the aortic pressure (red waveform) at rest.

Instantaneous wave-free ratio (iFR) is the first and the most-well established NHPR and was first introduced in 2012 (Figure 2) (34). The concept of iFR has been further validated against FFR (34, 35) and a cut-off of 0.89 was determined to be closely matched to the FFR threshold of 0.80 (35). Subsequently, two large randomised controlled trials (see Table 1) compared iFR and FFR with clinical outcomes as end-points and showed iFR-guided PCI was non-inferior to FFR-guided PCI (51, 52). In response, the 2018 European Society of Cardiology (ESC) guidelines on myocardial revascularisation were updated with a class 1a recommendation for both FFR and iFR in the assessment of intermediate grade stenoses when evidence of ischaemia is not available (53). Interestingly, although around 20% of cases showed discrepancy between FFR and iFR for borderline lesions at the respective thresholds of 0.8 and 0.89, this did not translate to difference in clinical outcomes (54). Any trial looking to definitively address this question was estimated to require 290,000 patients (55). In our view, when conducting invasive physiological assessment, there is no convincing difference between iFR and FFR: using either method is superior to angiography alone for the assessment of intermediate lesions.

**Table 1:** Comparison of invasive and Non-invasive physiological assessment tools (36–50, 63).

Hyperaemic pressure ratios	Strengths	Limitations	Validation	Ongoing trials
FFR	• Most validated invasive assessment against long-term clinical outcomes • Aids decision of OMT vs revascularization • Rationalizes coronary revascularization	• Invasive • Lengthy procedure • Adenosine hyperaemia induced side effects • Cost • Limited use in assessing serial lesions	• DEFER • FAME1,FAME2, • DINAMI3-PRIMULTI • COMPARE-ACUTE	• FAST III • FAST OCT • FAST STEMI II
CFR & IMR	• Assess microvascular function • Assess microvascular vasoreactivity • Aids in diagnosis of INOCA and ruling out angina	• Invasive • Lengthy procedure • Adenosine hyperaemia induced side effects • Risks of vasoreactivity testing • Cost	• CORMICA	• ICORMICA
Non-hyperaemic pressure ratios				
iFR	• Hyperaemia independent • Quicker than FFR • Low incidence of patient related discomfort • Validated in pullback analysis in tandem lesions • Low procedural cost	• Invasive • Epicardial assessment only • More sensitive to noise, hydrostatic effects and wire drift during pullback • More sensitive to variation of BP and heart rate	• ADVISE • ADVISE Registry • CLARIFY • DEFINE-FLAIR • SWEDEHEART	
RFR	• Similar to iFR	• No RCT • Invasive	• VALIDATE-RFR • RE-VALIDATE	
DFR	• Similar to iFR • Non inferior to iFR	• Insufficient validation • Lack of RCT • Limited availability of hardware & software • Not validated against FFR • Not validated in tandem lesions	• VERIFY 2 • CONTRAST • IRIS-FFR Registry	
QFR	• Low procedure time • Low cost • Can be used to assess tandem lesions • No pressure wire required	• Lack of RCT • Reduced accuracy compared to FFR • Requires trained and experienced operators • Availability of software • Not measurable in aortic-ostial lesions, severe tortuosity or overlapping vessels on angiogram • Not validated in bifurcation lesions	• FAVOR I, II	• FAVOR III
vFFR	• No pressure wire required • No pressure catheters required • Low interobserver variability	• Requires biplane imaging (30^o^ apart) • Requires additional training • Based on computational assumptions, not true measurements • Limited supporting evidence and lack of RCT	• FAST	FAST II
Non-invasive				
CT-FFR	• Non-invasive • Concomitant anatomic & physiological assessment • Cheaper than invasive FFR	• More expensive than other non-invasive tests • Impacted by artefact from stents, motion, misalignment • Not suitable for bypass grafts • Based on computational assumptions, not true measurements • Radiation exposure (albeit similar to invasive FFR assessment)	• DISCOVER-FLOW • NXT • FORECAST • SYNTAX III • DeFACTO	• Precise PCI Plan • DECISION
Stress echocardiography	• Wide availability • Non-invasive • No ionising radiation exposure • Relatively inexpensive • Not impacted by presence of stents or grafts	• Increased risk of arrhythmias with pharmacological stress • Dobutamine contraindicated in hypertension, significant LVOT obstruction or sustained ventricular arrhythmias • Suboptimal image quality due to body habitus, lung disease • Resting regional wall motion abnormalities or inter-observer variability limits diagnostic accuracy	• EVAREST • SPEED TRIAL	• ABCDE-(FGLPR) NCT05081115
MPI	• High sensitivity • Non-invasive • Can assess myocardial flow • Can assess cardiac function as well • Highly validated	• Long acquisition protocols • Less spatial resolution than other modalities • Comparatively high radiation • Prone to artefacts • Reduced sensitivity and specificity in multivessel disease	• ReASSESS • CREDENCE	
PET	• Non-invasive • Better diagnostic accuracy of CAD in women • Relatively lower radiation • High resolution, less attenuation artefacts and good image quality even in obese patients	• Limited availability • High cost	• PACIFIC	
MRI stress perfusion	• Non-invasive • Lack of radiation • High spatial resolution • Can perform absolute quantification of perfusion Provides addition information on cardiac structure and function Quantitative CMR perfusion measurements correlate well with FFR in in significant CAD	• High cost • Varying availability • Limited functional analysis in the presence of arrhythmias	• CE-MARC • CE-MARC 2 • MR-INFORM	

Both hyperaemic and non hyperaemic pressure ratios can be used in the post PCI setting but this is not routinely practiced. The evidence in this arena is rather equivocal. Although trials like the REPEAT-FFR showed that suboptimal physiological outcome (FFR < 0.90) was associated with a higher incidence of MACE at 1 year, other trials like FFR-SEARCH proved that post PCI FFR did not correlate with clinical events at 30 days (56, 57). However, the follow up period in FFR-SEARCH was considered too short and typically, clinically significant ISR would take months to manifest.

#### Other NHPR

Since the introduction of iFR, other NHPR have become available including Diastolic hyperaemia-Free Ratio (DFR) (Boston Scientific, MA) and Resting Full-cycle Ratio (RFR) (Abbott, IL). These are all proprietary and can only be used with the hardware and software provided by the vendors. They also have limited validation data and no randomised controlled trial (RCT) data to evaluate the clinical outcomes compared to established PCI strategies (see Table 1). They, however, generally do correspond well to iFR (58) with the VALIDATE-RFR retrospective study of 651 iFR waveforms finding that RFR correlated highly with iFR (*R*^2 ^= 0.985). Statistical equivalence testing within a 1% margin of error confirmed RFR and iFR were diagnostically equivalent (mean difference −0.002, 95% CI: = 0.009–0.006, *p* = 0.03) (59). It is widely accepted in the interventional cardiology community that these NHPRs are a reasonable substitute for FFR and iFR in most cases**.**

#### Angiography-derived FFR

Given the success of CT derived FFR, there is increasing interest in the development of novel non-invasive methods to assess FFR. Currently, 3 technologies are commercially available to assess FFR from coronary angiography alone: QFR (Medis Medical imaging systems, NE), FFR_angio_ (Cathworks IS), vFFR (Pie Medical, NE).

Quantitative flow ratio (QFR) has the most clinical data including large multi-centre trials and one RCT (60–63) (see Table 1). In the recent FAVOR III trial, QFR-guided PCI had significantly reduced 1-year MACE compared to angiography-guided PCI driven primarily by reduced MI and repeat revascularisation (63). There are less data for FFR_angio_ and vFFR: FFR_angio_ was recently validated in the prospective FAST-FFR study (64) whilst vFFR was validated retrospectively in a cohort study (65) (see Table 1). In a systematic review and Bayesian meta-analysis, angiography-derived FFR compared well to pressure wire FFR and no difference was demonstrated between online and off-line methods of analysis (66). One of the main advantages of angiography-derived FFR is the ability for rapid online and offline analysis. In the FAVOR II study, the median time for QFR computation was only 5 min, compared to 7 min for pressure wire FFR (62). Further randomised control trials are required before angiography-derived FFR can be considered as an alternative to pressure wire or CT-derived FFR.

### Computer tomography (CT) derived FFR

Most interventional cardiologists are familiar with the pressure wire study of coronary physiological indices such as FFR and instantaneous wave-free ratio (iFR). More recently, developments in computerised tomography (CT) have allowed the non-invasive estimation of FFR using fluid dynamics modelling. The most well-known and widely used CT derived FFR, i.e., FFR_CT_ (Heartflow, CA), is based on 3-dimensional coronary artery reconstruction and fluid dynamics. FFR_CT_ has been validated against and shown to be comparable to pressure wire FFR in three large prospective trials (3–5) (see Table 1) and further confirmed in a recent meta-analysis (6). In the PLATFORM and FORECAST trials, FFR_CT_ was shown to be feasible, safe and associated with lower rate of invasive coronary angiography with normal coronary arteries (7, 8). Furthermore, a negative FFR_CT_ (≥0.80) result is associated with good clinical outcomes at 12 months (9, 10) and 4.7 years from the follow up data of the NXT trial (11). This is currently being further evaluated in the ongoing randomised controlled PRECISE trial (NCT03702244). More recently, in the SYNTAX II trial, FFR_CT_ was demonstrated to be feasible in three vessel coronary disease and provided comparable results to pressure wire FFR (12). Based on these finding, SYNTAX III trial showed planning of revascularisation for patients with left main stem or three-vessel coronary disease based on CT and FFR_CT_ was feasible and correlated highly with decisions made from invasive coronary angiography (13). Growing evidence supporting FFR_CT_ for the evaluation and diagnosis of patients with chest pain is reflected in the guidelines. The National Institute for Health and Care Excellence (NICE) issued initial guidance for FFR_CT_ in 2017 as the most cost-effective recommended option, and this was updated in 2021 with expected savings of £9.4 million through avoidance of invasive tests and treatments (14).

In the American Heart Association/American College of Cardiology (AHA/ACC) guideline updated in 2021, CT coronary angiography (CTCA) is the recommended investigation for intermediate risk patients (1a recommendation) and FFR_CT_ is recommended for those with coronary stenosis of 40%–90% (2a recommendation) (15). A low risk anatomy is defined as either normal or stentosis <30%, for which FFR_CT_ is not required and optimal medical therpapy (OMT) would suffice as treatment. A high risk anatomy is defined as left main stem (LMS) disease of >50% stenosis, >70% stenosis in the LAD or three vessel stenosis for all of which and invasic coronary angiogram would be indicated, hence FFR_CT_ may be unnecessary. However, in this cohort, FFR_CT_ may be useful in patients with three vessel disease who are not fit for surgery and may benefit from PCI. This would also be applicable in patients with significant (>70%) single vessel disease and would aid in deciding between OMT and PCI. As mentioned above as well as reflective of the current guidelines, FFR_CT_ is most uself in the intermediate risk category (30%–69% stenosis) as it would help determine OMT vs. invasive coronary assessment and PCI.

A recent multicentre audit of clinical data with cost analysis of the use and efficacy of FFR_CT_ suggested that it had a low positive predictive value and costs £2,102 per patient compared with an average of £1,411 for stress imaging, making its use more expensive than conventional stress imaging modalities (16).

There are also other important limitations to consider for FFR_CT_. Sub-optimal imaging is a major limiting factor and often caused by factors such as breathing, fast or irregular heart rate. Even with improvement in technology, extensive coronary calcification remains a significant challenge. It is well known that coronary CT angiography (CTA) has low specificity and limited accuracy in the setting of increased calcification owing to calcium blooming and overestimation of luminal stenosis. FFR_CT_ values can potentially be affected as a result of calcium compromising the identification of vessel boundaries for modeling. Interstingly in a subset anlaysis of the NXT study and other machine learning techniques have demonstrated that discrimination of lesion spepcifc ischaemia as well as diagnostic accuracy and specificity of FFR_CT_ were higher at every level of calcium when compared to CTA alone (5). Hence, it may be valuable to perform FFR_CT_ in patients with extensive calcification particularly in planning revascularization of complex calcified lesions. FFR_CT_ lacks validation in revascularized vessels, microvascular dysfunction, spontaneous coronary artery dissection (SCAD), acute plaque rupture, coronary artery bypass grafts and congenital heart disease including coronary anomalies. Lastly, FFR_CT_ can only be performed in a central core lab (Heartflow, Redwood City, CA) which necessitates transfer of patient data and delay in analysis.

Other methods of CT derived FFR have been developed which allow real-time on-site analysis. Taking into account the limited available evidence, they all have acceptable accuracy, but they are not widely or commercially available currently.

### Microvascular disease & physiology

Many patients with stable angina undergoing coronary angiography are found to have myocardial ischaemia with non-obstructive coronary arteries (INOCA), the most common cause of which is coronary microvascular dysfunction (CMD).

The coronary microcirculation is a complex and structured system of small vessels (calibre <400 μm) which adapt their function in order to sustain the myocardium's physiological demands (67). Increasing evidence over the last few decades has shown that structural and functional abnormalities of the coronary microvasculature are highly prevalent and are associated with adverse clinical outcomes (68, 69).

Coronary Vasomotion Disorders International Study Group (COVADIS) formulated criteria (70) for the diagnosis of microvascular angina including:

(1) Angina secondary to myocardial ischemia
(2) Invasive or non-invasive evidence of unobstructed coronary arteries (<50% diameter reduction or FFR < 0.8)
(3) Objective evidence of myocardial ischemia (ETT, stress perfusion scan or RWMA on echo)
(4) Evidence of coronary microvascular function

Microvascular vasospasm fulfils the COVADIS criteria if the vasoreactivity test reproduces the usual anginal symptoms associated with ischemic ECG changes in the absence of significant epicardial spasm (67, 70).

The pathophysiology of CMD entails enhanced microvascular coronary vasoconstrictive reactivity, increased coronary microvascular resistance and impaired vasodilator capacity (68). Various pathophysiological mechanisms have been proposed. Impaired endothelium-dependent vasodilation affects the arterioles as a consequence of progressive endothelial dysfunction which leads to a reduction of nitric oxide production and release, hence resulting in insufficient nitric oxide mediated vasodilation (67). This is thought to be a consequence of loss of balance between myosin light chain kinase and phosphatase activity resulting in excess vascular smooth muscle contraction (71). Additionally, other factors like endothelial dysfunction, vascular smooth muscle hyper-reactivity, triggers like inflammation, oxidative stress and genetic factors are considered likely culprits of epicardial as well as microvascular spasm (67). Prognosis could be worse if there is associated unequivocal myocardial ischemia, and quality of life may be impaired due to recurrent angina, hospitalisation, and coronary angiography (72). A majority of patients with unobstructed coronary arteries have microvascular coronary abnormalities which can be safely investigated during coronary angiography. Invasive angiography can consequently provide vital diagnostic information using physiologic indices that can influence treatment and outcome (73).

The Coronary Microvascular Angina (CorMicA) trial assessed whether an interventional diagnostic procedure (IDP) linked to stratified medical therapy improved outcomes and quality of life in patients with INOCA (74). IDP entailed assessment of coronary vascular function and included guide wire-based assessment of coronary flow reserve (CFR), index of microcirculatory resistance (IMR), FFR and vasoreactivity (microvascular spasm) testing with acetylcholine. The authors conducted a randomised controlled blind clinical trial in which patients with INOCA were randomised to the intervention group or the control group (74). The primary endpoint was the mean difference in the severity of angina at six months which was assessed by the Seattle Angina Questionnaire summary score (74). CorMica ultimately confirmed that standard coronary angiography often failed to identify patients with microvascular disease (74). Conversely, IDP supplemented with optimal medical therapy improved angina in patients with INOCA without any major differences in major adverse cardiac events at six months (74).

### Microvascular assessment (CFR & IMR)

Coronary flow reserve and index of microcirculatory resistance are invasively calculated from two temperature sensors located on the proximal and distal portions of the intracoronary pressure wire, using thermodilution (75). Other non-invasive modalities such as transthoracic doppler echo, positron emission tomography and stress cardiac magnetic resonance (see Table 1) can also be used to determine CFR (67). The thermodilution method has, however, been proven to correlate well with true microcirculatory flow and resistance, and hence produces accurate and reliable CFR and IMR assessment (76).

The catheter is firstly flushed with 5 ml saline before a volume of 3 ml of ambient temperature saline is rapidly injected into the coronary artery. As it passes the proximal and distal sensors of the pressure wire, temperature changes are detected. The time interval of the passage of saline between the proximal and distal sensor is used to determine the coronary flow [mean transit time (Tmn)]. This process is done at rest as well as on induced hyperaemia.

Hyperaemia is induced with an intravenous infusion of adenosine at a dose of 140 mg/kg/min (77). Adenosine-mediated coronary microvascular tone and reactive hyperaemia occur through activation of adenosine receptors (especially A_2A_R) on endothelial as well as smooth muscle cells which results in coronary vasodilation (77). Other agents like intracoronary papaverine can be used at a dose of 12–16 mg for the left coronary artery and 8–12 mg for the right coronary artery. Intracoronary papaverine was previously the gold standard, but concerns have been raised due to its potential adverse effects of QTc prolongation and ventricular arrhythmias (78). Furthermore, the duration of action of papaverine is too short for steady state hyperaemic pressure recordings in comparison to intravenous adenosine which produces excellent steady state phasic intracoronary pressure recordings within one minute of initiation (78, 79). Adenosine is therefore the more ideal vasodilator of choice in epicardial and microvascular coronary physiology assessment.

CFR is calculated by dividing the rest Tmn by the hyperaemic Tmn. A CFR < 2.5 indicates impaired flow, at either the coronary or microvascular level. Based on Ohm's law, vascular resistance (R) is the pressure difference across the myocardium (*Δ*P = Pd-Pv) divided by the coronary flow rate (Q) which is inversely related to the Tmn (1/Tmn). Therefore R can be calculated as (Pd-Pv) × Tmn. Index of coronary microvascular resistance (IMR) is calculated with thermodilution as the product of the distal coronary pressures (Pd) and the Tmn during maximal hyperaemia (67). An IMR > 25 indicates high microvascular resistance and, indirectly, coronary microvascular dysfunction. Importantly, this measure is independent of the degree of coronary obstruction (67). A combination of a CFR < 2.5 and an IMR > 25 is diagnostic of microvascular dysfunction in patients with INOCA (80) (Table 2 and Figure 4). ESC guidelines on the management of chronic coronary syndromes (2019) recommend CFR and/or IMR should be considered in patients with persistent symptoms but have coronary arteries that are either angiographically normal or have moderate stenosis with preserved iFR and FFR (class 2a, level of evidence B). Similarly, the AHA/ACC guidelines for the evaluation and diagnosis of chest pain (2021) recommend consideration of invasive microvascular assessment in patients with persistent stable chest pain and INOCA (15) (class 2a, level of evidence B).

**Table 2:** Coronary pathophysiology based on microvascular physiology indices.

Coronary pathophysiology	Hyperaemic physiology (Adenosine)	Acetylcholine test
Microvascular dysfunction (MVD)	CFR < 2.5, IMR > 25	No ischemic symptoms or ECG changes
Microvascular spasm	CFR < 2.5, IMR < 25	Ischemic symptoms or ECG changes
MVD and microvascular spasm	CFR < 2.5, IMR > 25	Ischemic symptoms or ECG changes
Negative study (Figure 3)	CFR > 2.5, IMR < 25	No ischemia

**Figure 3:**
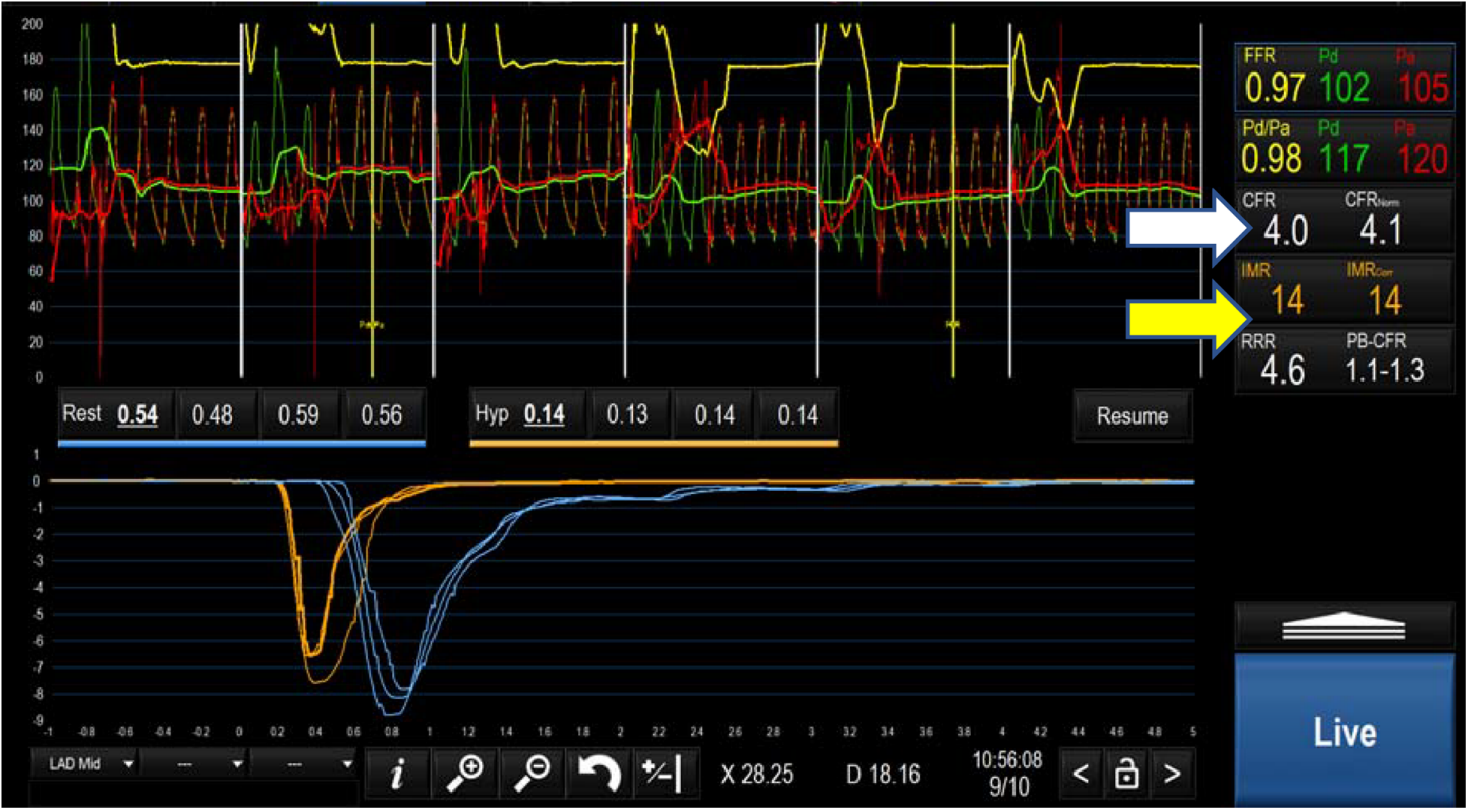
Negative microvascular study demonstrating a CFR of 4 (white arrow) and an IMR of 14 (yellow arrow) ruling out microvascular dysfunction.

**Figure 4:**
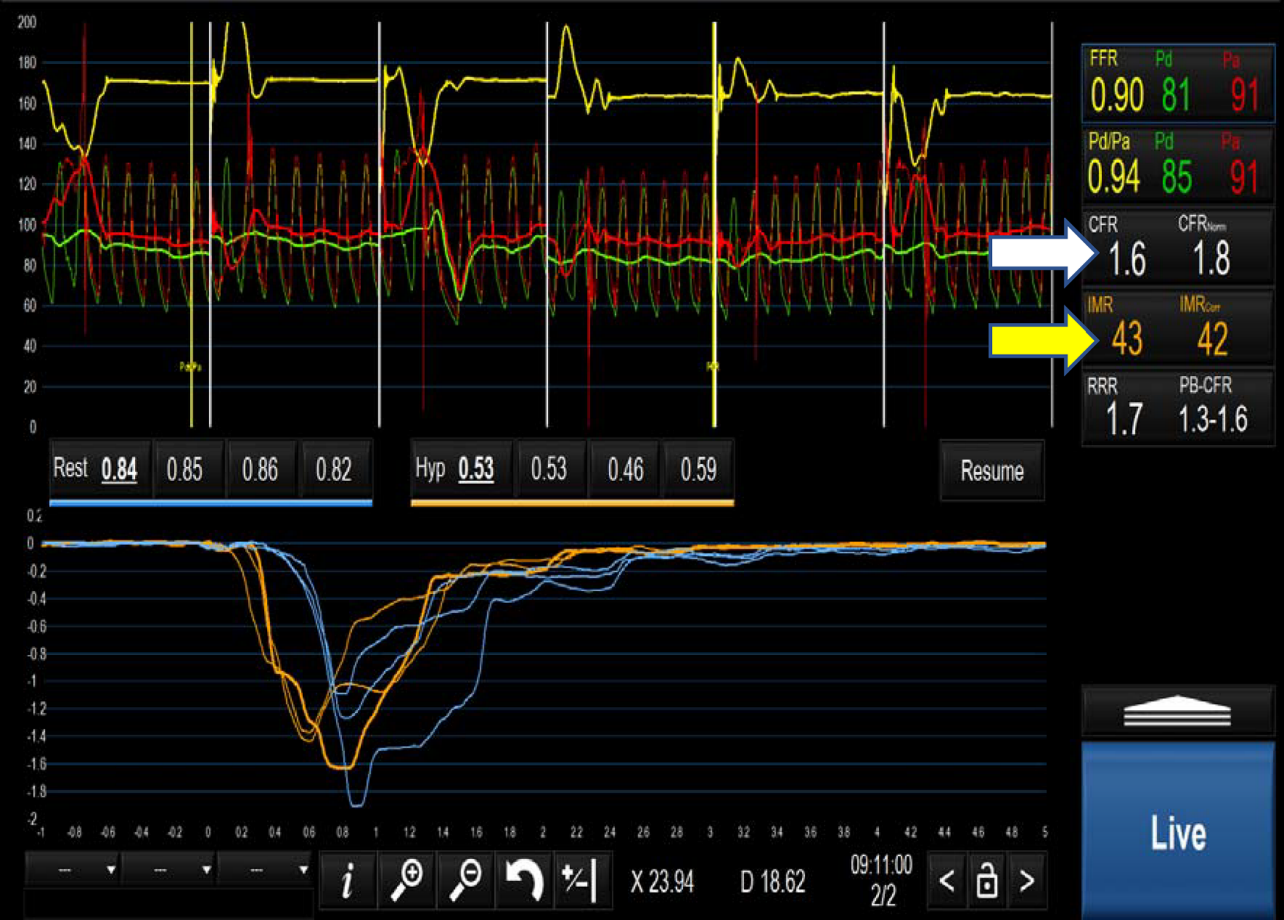
Positive microvascular study demonstrating a CFR of 1.6(white arrow) and an IMR of 43 (yellow arrow) fulfilling the criteria for MVD i.e. CFR < 2.5 and IMR > 25.

### Vasoreactivity testing

When microvascular spasm is suspected, usually a provocation test is performed by administering intracoronary acetylcholine (Ach) (67, 81). High doses of intracoronary acetylcholine are administered, acting on both the epicardial coronary arteries as well as the coronary microvasculature, which unmasks any underlying vasomotor abnormalities hence resulting in vasoconstriction rather than vasodilation (67, 71). As per the COVADIS criteria, a positive microvascular vasoreactivity test would reproduce ischemic symptoms as well as ECG changes in the absence of significant epicardial spasm (<90% coronary diameter reduction) (70). If however, there is >90% coronary diameter reduction, a diagnosis of coronary vasospasm is evident.

Acetylcholine 20–100 mcg is usually administered in a stepwise manner into the left coronary artery (LCA) and 20–50 mcg into the right coronary artery (RCA) over a period of 20 s with a 3–5 min interval between each injection. In view of the potential adverse consequences of acetylcholine, such as ST elevation myocardial infarction, chest pain, bradyarrhythmias and ventricular tachycardia and fibrillation (82, 83), this test is not universally offered and our centre does not routinely practice vasoreactivity testing. Consideration should be given to transferring the patient for assessment at a specialist unit. Other agents that can be used are ergonovine (ER), neuropeptide Y and dopamine. However the vast majority of data supports the clinical use of predominantly Ach as well as ER (71).

### Treatment strategies

**Figure 5:**
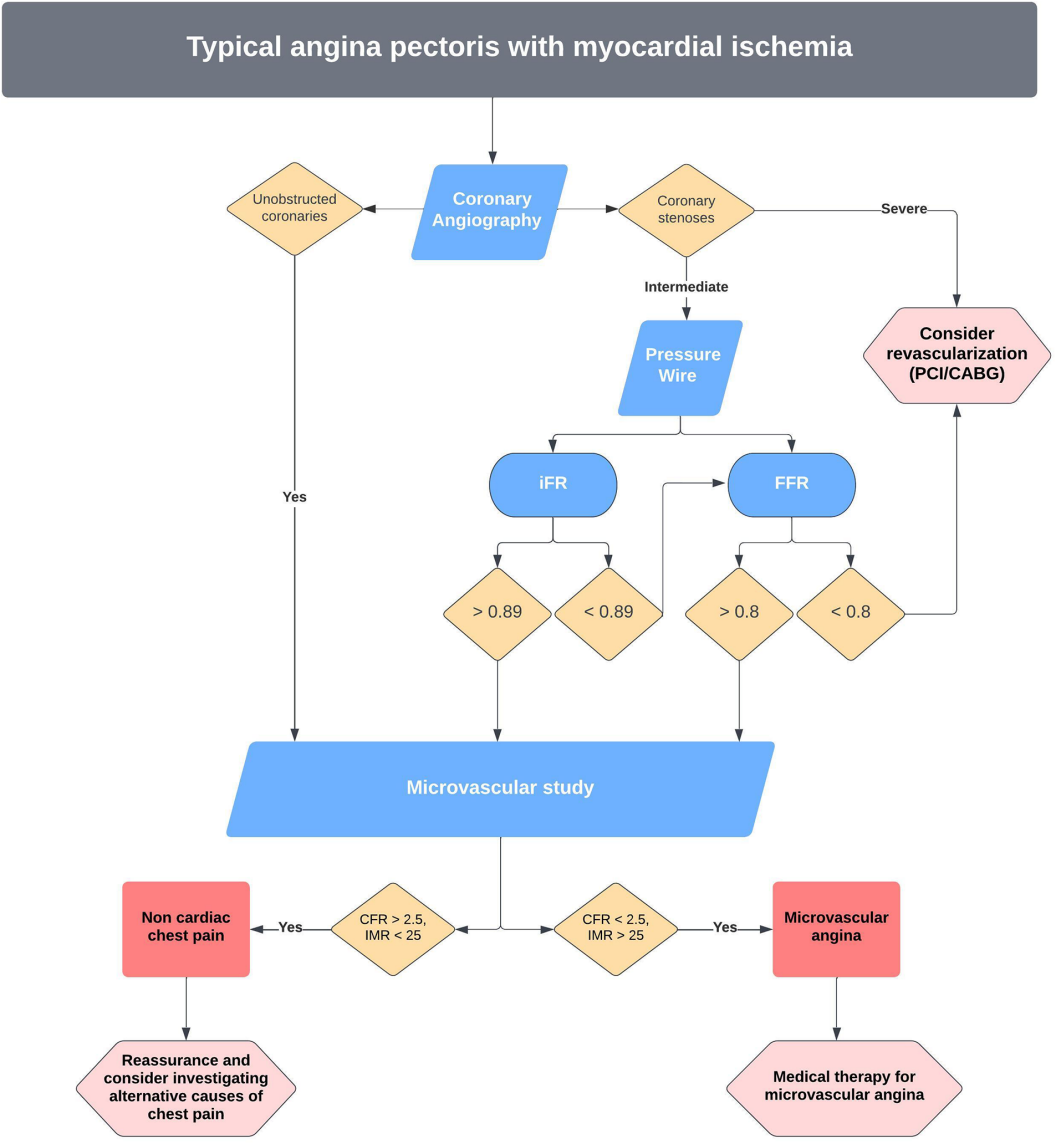
Proposed pathway for invasive management of angina and microvascular dysfunction at our centre.

The treatment of microvascular dysfunction is dependent on the cause. If the aetiology is impaired microcirculatory conductance, (CFR < 2.5, IMR > 2.5), empirical anti-anginal therapy is recommended acknowledging limited trial data (Figure 5). Current European Society of Cardiology (ESC) guidelines recommend beta-blockers as the first line therapy in addition to an ACE-inhibitor and statin. Weight loss and lifestyle changes should also be recommended. Short acting nitrate agents may help to relieve symptoms during an attack and if effective, adjunctive long-acting nitrates may also be given. In cases of microvascular dysfunction caused by a microvascular vasomotor disorder, calcium channel blockers and long-acting nitrates are the preferred treatment (84).

## Conclusion

Invasive coronary physiology assessment is proving to be a valuable adjunct to coronary angiography in aiding the diagnosis of physiologically significant epicardial and microvascular coronary artery disease. While FFR (and increasingly NHPR) assessment of intermediate epicardial stenoses has become widely adopted, routine coronary physiology evaluation in INOCA patients is not commonplace, despite the low risk and straightforward methodology. This is partly due to the additional training, procedural time and cost involved. However, these disadvantages are likely to be offset by the ability to offer definitive diagnoses and appropriate treatments, thereby reducing recurrent hospital admissions and repeated investigations.
